# Vietnam's foreign policy (1945-1946): Proactive in a fragile independence

**DOI:** 10.12688/f1000research.166625.1

**Published:** 2025-07-03

**Authors:** Quoc Dung Mai

**Affiliations:** 1 Ho Chi Minh City University of Industry and Trade, Ho Chi Minh, Vietnam

**Keywords:** Foreign policy, Proactive, Independence, Fragile;, 1945-1946

## Abstract

**Background:**

The August Revolution of 1945 established the Democratic Republic of Vietnam, but the nascent government immediately faced immense challenges from external forces like French colonialists and Chiang Kai-shek’s army, alongside internal difficulties. In this precarious situation, diplomacy emerged as a crucial strategic tool for the Vietnamese revolution during the 1945-1946 period, demonstrating a skillful blend of struggle and negotiation to safeguard independence.

**Methods:**

This study is grounded in the principles of dialectical and historical materialism, consistent with the viewpoint of the Communist Party of Vietnam. The main methods include: the historical method (systematic examination of events and policies), the logical method (reconstructing diplomatic strategies), intertextual analysis (comparing Party directives with diplomatic actions), critical discourse analysis (Ho Chi Minh’s statements), and comparative assessment (with other decolonization movements). Data were collected from declassified archival materials, legal texts, diplomatic records, and contemporary press.

**Results:**

During 1945-1946, Vietnam implemented an independent, self-reliant, and open foreign policy based on principles of equality and mutual assistance, with the core objective of protecting independence, sovereignty, and territorial integrity. This policy demonstrated strategic flexibility by conciliating Chiang Kai-shek’s forces to free up resources against the French, and by signing the Preliminary Accord and Provisional Agreement with France to gain time for resistance preparation. Vietnamese diplomacy also proactively established friendly relations with neighboring countries and major powers, sought international recognition, and committed to multilateral cooperation, thereby strengthening the legitimacy of the revolutionary government.

**Conclusion:**

In an extremely challenging situation, foreign affairs activities, under the leadership of the Party and President Ho Chi Minh, successfully protected Vietnam’s independence and enhanced the Democratic Republic of Vietnam’s prestige. The strategic lessons on foreign policy thinking from 1945-1946 have become a firm foundation for Vietnam’s modern foreign policy. They emphasize the harmonious combination of national independence, socialism, genuine patriotism, and internationalism, striving towards the ultimate goal of building a “prosperous people, strong country, democracy, justice, and civilization” in Vietnam.

## Introduction

Following the August Revolution, Archimedes L.A. Patti observed that “Hanoi alone became something like a center of underground and mysterious international movements,” with journalists and agents from “the U.S., Britain, France, China, the Netherlands, India, and the Soviet Union” converging on the city ( Archimedes L.A.Patti, 2008, p. 577). Despite divergent agendas, imperialist powers and foreign reactionaries shared a singular objective: the dismantling of Vietnam’s nascent revolutionary government and the reversal of the achievements of the August Revolution.

This paper examines the socio-political and cultural transformations in Vietnamese society that originate in 1945 and were rooted in early 19th-century dynamics. From the proclamation of independence and the founding of the Democratic Republic of Vietnam (DRV), the country has endured a tumultuous trajectory: two devastating wars, a national partition, and profound shifts in political and social structures. Across this period, new generations emerged, cultural values underwent adaptation (or rejection), and societal norms evolved a process that is still unfolding today. Notably, the Communist Party of Vietnam (CPV), established by Ho Chi Minh in the 1930s, persisted as the state’s unchanging leadership force. Since 1945, the CPV has functioned as the sole architect and guarantor of Vietnam’s most critical societal transformation ( Novakova O.V., 2015, p. 240-261).

For smaller nations confronting imperial domination, total struggle encompassing diplomacy is imperative. Drawing on his global revolutionary experience, Hồ Chí Minh, leader of Vietnam’s asymmetrical resistance, asserted: “Whoever seizes the diplomatic advantage will win” ( Ho Chi Minh 2011a, p. 559). His flexible yet strategic foreign policy during 1945–1946 exemplifies this principle by securing critical leverage for the revolution.

### Vietnam’s Civilized and Ethical Diplomacy: A Testament to Maturity and Progressive Values

Following the August Revolution, Vietnam faced multifaceted challenges, yet its foreign policy demonstrated remarkable civilizational maturity, compelling adversaries to acknowledge its legitimacy. As Dixee R. Bartholomew-Feis (2007, p. 357) notes, Vietnam’s diplomacy shattered colonial stereotypes, disproving the notion that the nation was “barbaric,” as French propaganda had long claimed. This sentiment was echoed by American journalist Panlo Hop, who, after visiting Vietnam in December 1945, affirmed that the Vietnamese people were a civilized nation deserving international recognition for their independence ( Ho Chi Minh 2011b, p. 151).

The failure of France’s Instruction on December 10, 1946 which omitted any reference to its supposed “civilizing mission” further underscored Vietnam’s political and cultural autonomy ( Stein Tonnesson, 2013, p. 296). This marked a decisive rejection of colonial rhetoric, positioning Vietnam as a democratic and progressive state fully capable of standing alongside other sovereign nations.

Hồ Chí Minh, Vietnam’s revolutionary leader, articulated the nation’s diplomatic philosophy with clarity: “We must rely on strength. Strong strength and diplomacy win. The strength is like a gong, and diplomacy is its resonance. The louder the gong, the farther its sound carries” ( Ho Chi Minh 2011c, p. 147).

This principle emphasized soft power as the cornerstone of Vietnam’s post-revolutionary diplomacy rooted in progressive cultural-ethical values and aligned with the nation’s historical and material conditions. From a dialectical-materialist perspective, Vietnam’s diplomacy was both a product and a driver of an advanced socio-political environment, one that upheld universally recognized legal and humanitarian principles.

Ho Chi Minh’s vision extended beyond resistance: he sought global solidarity for peace and shared prosperity. While condemning imperialist violations of the Atlantic Charter and the San Francisco Charter, he simultaneously advocated for Vietnam’s territorial integrity and peaceful coexistence ( Bui Dinh Phong, 2021). This dual approach, steadfast in its sovereignty yet open to equitable international partnerships, is a prime example of Vietnam’s civilized and ethical statecraft, distinguishing it from the very forces that have denied Vietnam its right to self-determination.

### Vietnam’s Commitment to International Cooperation and Multilateral Engagement

Upon declaring independence, Vietnam proactively communicated its openness to global cooperation, addressing the French government, allied powers, and the United Nations with a clear message: Vietnam was a sovereign nation ready to collaborate in nation-building and global progress. As Ho Chi Minh articulated, “Any country including France that sincerely wishes to invest in Vietnam for mutual benefit will be warmly welcomed” ( Ho Chi Minh 2011c, p. 145). He further emphasized Vietnam’s inclusive approach: “We will invite French experts, as well as American, Russian, or Chinese specialists, to assist in our national reconstruction” ( Ho Chi Minh 2011b, p. 86).

Vietnam actively supported the creation of the Far East Advisory Committee, asserting its eligibility to appoint representatives and contribute to resolving regional challenges. This demonstrates Vietnam’s commitment to institutional diplomacy and its belief in collective problem solving. Ho Chi Minh’s visionary leadership transcended national liberation; his ideology, rooted in a progressive worldview, became not only a guiding doctrine for Vietnam’s Communist Party but also a significant contribution to global political thought ( Selivanov I.N., 2021).

To secure international recognition, Vietnam’s diplomacy leveraged allied wartime commitments, particularly the principles of self-determination and equality among nations. President Ho Chi Minh engaged in high-level correspondence with leaders of the U.S., the UK, the Soviet Union, China, and the UN General Assembly, formally announcing Vietnam’s independence and seeking support.

Recognizing the ambiguous U.S. stance on Indochina, Vietnam strategically cultivated ties with American representatives including the U.S. Mission in Indochina and the Office of Strategic Services (OSS) to counterbalance French colonialists and Chiang Kai-shek’s forces. These efforts persuaded the U.S. to adopt a neutral position, mitigate external pressures and buy Vietnam’s critical time to consolidate its governance ( Dang Dinh Quy, 2016).

Vietnam’s cultural diplomacy following the August Revolution (1945) yielded significant outcomes, projecting the nation’s identity globally, reinforcing broader foreign policy efforts, and establishing a crucial foundation for the subsequent resistance struggle ( Nguyen Thanh Tung, 2018). This period marked a transformative phase in which Vietnam’s diplomatic strategies were deeply intertwined with its revolutionary ethos and the quest for international legitimacy.

This article examines the formation and evolution of Ho Chi Minh’s worldview, shaped over decades of revolutionary activism and the protracted struggle for Vietnamese independence. As Nguyen Thi Bich Ngoc and Shpkovskaya (2022, p. 281-300) highlight, his ideological framework forged through global exposure (Europe, Asia, and America) and anti-colonial resistance became the bedrock of Vietnam’s foreign policy.

### While prior research has outlined the general characteristics of Vietnam’s 1945–1946 foreign policy, critical aspects remain underexplored

Foreign Policy Objectives: Was primary goal of securing immediate recognition or long-term strategic alliances?

Guiding Principles: How did independence, flexibility, and anti-colonial solidarity shape diplomatic tactics?

Implementation Strategies: What differentiated Vietnam’s approach from other post-colonial states?

This article addresses these gaps and, offers a systematic analysis of Vietnam’s early diplomacy in order to clarify its strategic coherence and historical significance.

## Methods and data

This study is grounded in the principles of dialectical and historical materialism, adhering to the ideological perspective of the Communist Party of Vietnam and the Socialist Republic of Vietnam. This study employed a dual methodological approach, integrating the following:

*The Historical Method:* A systematic examination of events, policies, and diplomatic actions within their temporal and socio-political contexts, tracing the evolution of Vietnam’s foreign policy from 1945 to 1946.

*The Logical Method:* An analytical reconstruction of diplomatic strategies to reveal their underlying principles, causal relationships, and strategic coherence.

*Intertextual Analysis:* Cross-referencing the Communist Party of Vietnam directives with diplomatic actions to assess policy implementation.

Critical Discourse Analysis: Examining rhetorical strategies in Ho Chi Minh’s letters/public statements to discern diplomatic narratives.

*Comparative Assessment:* Contrasting Vietnam’s approach with other post-1945 decolonization movements (e.g., Indonesia, India) to identify unique adaptations of Marxist-Leninist principles.

The methodology aligns with Marxist historiography by emphasizing class struggle and anti-imperialism as drivers of Vietnam’s foreign policy, treating diplomacy as a superstructure phenomenon shaped by material conditions (e.g., post-war economic devastation and colonial legacies), and evaluating the dialectical interplay between Vietnam’s revolutionary objectives and global power structures.

The research draws extensively from declassified archival materials, now accessible in published formats, including the Communist Party of Vietnam Documents, Central Committee resolutions, Politburo directives, and Communist Party of Vietnam Congress reports on foreign policy; Legal and Governmental Texts: Decrees, regulations, and diplomatic circulars issued by the Provisional Government of the Democratic Republic of Vietnam; Diplomatic Records: Official statements, memoranda, and correspondence between Vietnamese leaders (e.g., Ho Chi Minh, Phạm Van Dong …) and foreign governments/institutions (U.S., France, USSR, UN); Periodicals and Press Archives: Contemporary newspapers (e.g., Cuu Quoc, Su That …) and international press coverage to gauge global perceptions of Vietnam’s diplomacy.

## Results and Discussion

### Foreign policy in the years 1945-1946

The successful August Revolution in 1945 led to the establishment of the Democratic Republic of Vietnam. The Declaration of Independence, proclaimed on September 2, 1945, unequivocally stated: “The Provisional Government of Vietnam, representing the entire Vietnamese people, declares complete separation from colonial relations with France, annulling all treaties France imposed on Vietnam and abolishing all French privileges in Vietnamese territory” ( Ho Chi Minh 2000a, p. 3). The document further affirmed: “Vietnam has the right to enjoy freedom and independence, and has in fact become a free and independent nation. The Vietnamese people are determined to mobilize all their spiritual and material resources their lives and property to safeguard these fundamental rights” ( Ho Chi Minh 2000a, p. 4). This historic declaration not only marked Vietnam’s independence but also established the principle that only sovereign nations possess the right to determine their own foreign policies.

The nascent government faced an extremely precarious situation: Vietnam’s independence lacked international recognition; over 30,000 allied troops (including French colonial forces and Chiang Kai-shek’s army) were stationed in the country, with both groups actively working to overthrow the revolutionary government; Viet Minh possessed only about 80,000 poorly equipped troops; opposition parties (Viet Quoc and Viet Cach), backed by foreign powers, controlled half of the Provisional Government’s ministries.

The nation confronted severe challenges: an empty national treasury, the lingering effects of the 1945 famine that claimed over two million lives, a 90% illiteracy rate, and extremely low agricultural productivity (about 12 quintals of rice/ha).

These overwhelming difficulties threatened the collapse of the fledgling government. Using any conventional measure of material strength, the Democratic Republic of Vietnam was destined for rapid collapse. However, through strategic methods of struggle including innovative diplomatic efforts Vietnam gradually overcame these challenges, laying the groundwork for the subsequent resistance war against the French colonial forces.

*Regarding foreign affairs,* under the new conditions of direct leadership of the government, the Party outlined internal and external policies to serve as the cause of resistance war and national construction to victory. Foreign affairs are placed in an important position with a system of viewpoints, strategies, and tactics on Vietnam’s relations with the world.

On October 3, 1945, the Provisional Government of the Democratic Republic of Vietnam issued a “Communication on the foreign policy of the Democratic Republic of Vietnam,” which clearly stated that Vietnam’s foreign policy was built on the basis of Vietnamese practice and the international situation. This means that the Vietnamese people themselves draw up an independent foreign policy and direction on the basis of the requirements and tasks of the Vietnamese revolution, but at the same time must be in line with international standards, appropriate corresponding to the trend of the times.

Vietnam’s foreign policy goal is to contribute to “bringing the country to complete and permanent independence.” It is a consistent affirmation of the foreign policy mission to ensure the interests of the nation and the nation, and to ensure basic national rights such as national independence, national sovereignty, territorial integrity, and unity.

The communiqué mentioned Vietnam’s foreign policy with a number of key subjects in international relations such as: major countries, countries in the anti-fascist allies, “Vietnam is extremely friendly and sincerely cooperates with on the stance of equality and mutual love”; “particularly for the French government, which advocates domination of Vietnam, it resolutely opposes it”; with neighboring countries, the communiqué emphasizes friendship, cooperation and equality; With the two countries of Cambodia and Ai Lao, Vietnam advocates that “the line of communication with the nation’s self-determination as the foundation must be even closer”…

*Regarding foreign policy principles,* Vietnam’s diplomacy takes the principles of the Atlantic Charter as the foundation, and the directive of the Central Executive Committee on the National Resistance War dated November 25, 1945 stated: “persist with the diplomatic relations with other countries on the principle of equality and mutual assistance. Particular attention must be paid to these: one is that diplomacy is to make one’s country fewer enemies and more allies than most; second, for diplomacy to be successful, we must show our strength” ( Communist Party of Vietnam, 2000b, p. 27).

Upholding the goals and principles, and at the same time willingness to implement an open foreign policy is a unique feature of Vietnam’s new foreign policy. In his Call to the United Nations, Ho Chi Minh clearly stated the foreign policy of the Democratic Republic of Vietnam: “In its foreign policy, Vietnamese people adhere to the following principles: 1. As for Laos and Myanmar, Vietnam respects the independence of these two countries and expresses its desire to cooperate on the basis of absolute equality between sovereign countries: 2. For democratic countries, Vietnam is ready to implement an open-door policy and cooperate in all fields: a) Vietnam won a favorable reception for investment from capitalists and engineers in all its industries. b) Vietnam is ready to expand its ports, airports and roads for international trade and transit. c) Vietnam accepts joining all international economic cooperation organizations under the leadership of the United Nations. d) Vietnam is ready to conclude with the navy and army forces within the framework of the United Nations special security agreements and treaties relating to the use of some naval bases and airspace ( Ho Chi Minh, 2000a, p. 469-470).

*Regarding the foreign policy motto,* Vietnam’s diplomacy has thoroughly grasped the viewpoints of independence, self-reliance, and self-reliance. In international relations, it is necessary to grasp the motto of being persistent in principle, firm in strategy, but flexible and flexible in strategy: “Our unchanging goal is still peace, unity, and independence establishment, democracy. Our principles must be firm, but our strategies must be flexible” ( Ho Chi Minh, 2000b, p. 319).

The Party’s foreign policy motto shows its proactive, positive and self-reliant stance; releases me by my strength; “If we are strong, then they will care about us. If we are weak, we are just an instrument in the hands of others, even if that person can be our ally” ( Communist Party of Vietnam, 2000a, p. 244).

### The results of the implementation of the policy

Vietnam’s new foreign policy has been effective since the beginning of its revolutionary government. In the years 1945-1946, the Vietnamese revolution had to deal with many dangerous enemies. On the basis of correctly determining “Our main enemy at this time is the invading French colonialists, must focus the fire of struggle on them, the Party has implemented a clever foreign policy: at times advocated “friendly Sino-Vietnamese”, détente with Chiang Kai-shek to limit their actions, so that they would not oppose the Vietnamese revolution, but leave their hands free to deal with the French colonialists, sometimes making peace with the French to push Chiang’s troops back home, implementing the policy of “reconciling peace and harmony” to achieve the goal”. These are examples of flexibility in strategy and ingenuity in taking advantage of conflicts between hostile forces to bring the Vietnamese revolution into a dangerous situation.

The fact that President Ho Chi Minh on behalf of the government of the Democratic Republic of Vietnam signed with the representative of the French Government the Preliminary Agreement on March 6, 1946 and the Provisional Agreement on September 14, 1946, the optimal foreign policy solution to protect the achievements of the revolution, taking advantage of more time to prepare for the long-term resistance war against the French, which our Party knows is inevitable.

Regarding Southeast Asian countries. For Laos and Cambodia, the policy of the Communist Party of Vietnam is: “Unifying the Vietnam - Cambodia - Laos front against aggression”. On October 30, 1945, the Agreement on Military Alliance between the Government of Itxata and the Government of the Democratic Republic of Vietnam along with the Agreement on the Alliance of Laos, Vietnam was signed and began to be implemented. For Asian countries, Vietnam actively opens friendly relationships. Immediately after the birth of the Democratic Republic of Vietnam, the Government sent a representative to Bangkok (Thailand) to enlist the support of the government and the people of the country. The Vietnamese Government’s special envoy held meetings with diplomatic representatives of India and Indonesia, creating the basis for Vietnam’s relations with these countries.

In fact, immediately after winning power and establishing a new Vietnamese State, the Party was rightly aware of the position and role of foreign affairs in the resistance war and national construction. On that basis, the Party soon established an independent and self-reliant foreign policy, including the following: foreign affairs objectives and tasks, arrangement of forces, determination of principles, mottos and methods of diplomatic struggle of the Vietnamese revolution. The new Vietnamese state’s foreign policy “renovated the relationship between the country and its colony and its neighbors near and far - including relations with major countries, opening a new history in international relations” ( Nguyen Phuc Luan 2001, p. 82).

Before and after independence, as the head of the Provisional Government, President Ho Chi Minh sent many emails and letters to the heads of state and foreign ministers of countries and organizations such as the United States, China, the Soviet Union and the United Nations. The letters and telegrams show Vietnam’s diplomatic views; that is, from the beginning, it has tried to expand relations with other countries, especially great powers, to enlist the recognition of Vietnam’s legal status. The independent South, thereby establishing a host position in communication with foreign countries, protects the newly established democratic republic. However, in the complicated context of the world situation at that time, the Vietnamese people had to fight against French colonialists and protect the revolutionary achievements in an almost lonely situation. However, while the fledgling revolutionary government was in a situation of “thousands of pounds hanging by a hair,” enemies inside and outside, facing difficulties on all sides, Vietnam’s foreign affairs had successfully completed its tasks, contributing to protecting and strengthening the revolutionary government, preparing forces for a long resistance war, and leaving valuable lessons for foreign affairs in the current period.

The period 1945-1946 was the most special and meaningful period in the nation’s history, and this was also a memorable period of Vietnamese diplomacy. With the correct, flexible and resolute policies and measures of President Ho Chi Minh, he created opportunities to take advantage of them to win.

That diplomatic strategy left behind extremely valuable lessons, not only meaningful during the years of fighting the French and the Americans but also meaningful today.

## Some lessons learned

### First, the lesson emphasizes the legitimacy and strength of the revolutionary government

In August 1945, when the Second World War ended, the global situation changed at an extremely rapid pace. Thanks to correctly predicting the world and domestic situation, knowing that Japan was about to surrender to the Allies and that the Japanese army in Vietnam was extremely confused, the Indochina Communist Party decided to seize the opportunity and launch a General Uprising to seize power. The Party advocates that it must gain power and declare independence before the allied troops enter, promote the position of the Democratic Republic of Vietnam, gain legal status for the new government, and take advantage of international recognition to facilitate transactions with allies.

To create a legal basis and official name for the new government, the Revolutionary Command, which had just returned to Hanoi, decided to reform the National Liberation Committee of Vietnam into the Provisional Government of the Democratic Republic of Vietnam. On August 28, 2945, some units of the Republic of China’s army began to move into North Vietnam, and an independence declaration ceremony was held before Chiang’s army arrived in Hanoi. On September 2, 1945, President Ho Chi Minh read the Declaration of Independence on the nation and the world, affirming that Vietnam has the right to enjoy freedom and independence and has truly become a free and independent country…. the entire Vietnamese people are determined to use all their spirit and force, their lives and property, to maintain that right to freedom and independence…

After declaring independence on September 3, 1945, the government held its first session, proposing six major tasks, including organizing the National Assembly election as soon as possible throughout the country. On January 6, 1946, the first General Election in Vietnam’s history was held successfully. On March 6, 1946, Vietnam signed a Preliminary Agreement with France. Thus, in just a short time, thanks to the strength of solidarity of the entire nation, Vietnam established a completely legal and constitutional government, representing all Vietnamese people to perform their functions. President Ho Chi Minh also invited former Emperor Bao Dai to join the new government as a Government Advisor, continuing to add to the government apparatus many former Ministers of the Nguyen Dynasty; therefore, Vietnam the new South wants to tell the world that the key elements of the old regime all recognize and cooperate with the new regime.

On October 3, 1945, one month after declaring independence, the Ministry of Foreign Affairs of the Provisional Government issued a communiqué on foreign policy of the Democratic Republic of Vietnam, affirming the goal of striving for complete independence. The Communiqué was the State’s first document on foreign affairs, orienting the Party’s foreign affairs activities during the resistance war to build the nation, but first of all, to take advantage of and create momentum with allied forces in Vietnam. In the last few months of 1945 and early 1946, President Ho Chi Minh also sent many letters and diplomatic notes to major countries such as the US, UK, Soviet Union, China and the President of the United Nations General Assembly announcing life and affirming the legitimacy of the Democratic Republic of Vietnam, denouncing France’s return to invade Indochina.

It can be said that the above-mentioned diplomatic strategies have contributed to enhancing the legitimacy and strength of the revolutionary government to confront aggressive forces, create the ability to add friends and reduce enemies, and create favorable conditions for foreign countries. Activities of the young revolutionary government.

### Second, the lesson on how to distinguish enemies

After the success of the August Revolution, a serious challenge for the Vietnamese revolution was simultaneously dealing with many opposing military forces in major countries present in Vietnam at the same time.

The skillful and correct strategies mentioned above are a novel step in Vietnam, appeasing the opposition of the Republic of China and Vietnam, contributing to the prevention of many sabotage and subversion plots of the enemy but still ensuring the principle of maintaining a strong government in Vietnam.

It can be said that the distinction between “friends and enemies” in the previous period was the basis for the Vietnamese Party to form the viewpoint of “partner and object” in the period of international integration.

### Third, the lesson of knowing how to make concessions at the right time, make concessions with limits, and make concessions with principles

On September 2, 1945, the Democratic Republic of Vietnam was born and faced many difficulties in economics, politics, culture, security, and defense. Faced with this situation, in terms of diplomacy, the Party and the Government have implemented a policy of temporary concessions and peace while still ensuring the principles of national independence and sovereignty.

In the current period, the world and regional situation have undergone many unpredictable changes, making Vietnam’s defense and homeland protection, especially maritime security, a difficult and challenging task. From the historical lessons mentioned above, the Party and the State of Vietnam have inherited and creatively applied them to foreign affairs activities during the new period.

## Conclusion

After the August Revolution in 1945, Vietnam faced an extremely difficult situation that seemed insurmountable, but under the leadership of the Party and President Ho Chi Minh, the foreign activities of the state opened a diplomatic struggle situation, making diplomacy an important part of the resistance line, contributing to protecting national independence and prestige of the Democratic Republic of Vietnam.

The lessons of strategic significance in foreign thinking in the years 1945-1946 are the foundation for Vietnam’s foreign policy guidelines today. The national interests must be placed in the relationship and harmoniously combined between national independence and socialism, between true patriotism and internationalism, for the highest goal is national construction Vietnam “rich people, strong country, democracy, justice, and civilization”. This will also be the basis for the formation, development and comprehensive implementation of Vietnam’s foreign policy of independence, self-reliance, openness, multilateralization and diversification of international relations.

## Ethical considerations

No ethical approval or consent was required.

## Data Availability

The data used in this study were obtained from secondary sources. Raw data were not collected and used in this particular article.

## References

[ref6] Bartholomew-Feis DR : *OSS and Ho Chi Minh – Surprise allies in the war against Japanese fascists.* Hanoi: World Publishing House;2007.

[ref3] Communist Party of Vietnam: *The Complete Party Document.* Vol.7. Hanoi: National Political Publishing House;2000a.

[ref4] Communist Party of Vietnam: *The Complete Party Document.* Vol.8. Hanoi: National Political Publishing House;2000b.

[ref7] Ho Chi Minh: *Complete volume.* Vol.4. Hanoi: National Political Publishing House;2000a.

[ref8] Ho Chi Minh: *Complete volume.* Vol.7. Hanoi: National Political Publishing House;2000b.

[ref9] Ho Chi Minh: *Complete volume.* Vol.3. Hanoi: National Political Publishing House;2011a.

[ref10] Ho Chi Minh: *Complete volume.* Vol.4. Hanoi: National Political Publishing House;2011b.

[ref11] Ho Chi Minh: *Complete volume.* Vol.6. Hanoi: National Political Publishing House;2011c.

[ref13] Ngoc NTB Shpakovskaya MA : Ho Chi Minh’s worldview and Vietnam’s foreign policy during the period of renewal. *Southeast Asia: Current Problems of Development.* 2022;4(4):281–300. 10.31696/2072-8271-2022-4-4-57-281-300

[ref14] Nguyen Phuc Luan: *Modern Vietnamese diplomacy for the cause of independence and freedom (1945-1975).* Hanoi: National Political Publishing House;2001.

[ref15] Nguyen Thanh Tung: Ho Chi Minh with the construction of Vietnamese cultural diplomacy in 1945-1946. *Foreign Relations Journal - Central Committee for External Relations.* 2018;4(102). 1859-2899.

[ref16] Novakova OV : Independent Vietnam: democracy versus traditionalism: 1945-1946 - the early XXI Century. *The Russian Journal of Vietnamese Studies.* 2015;1(5):240–261.

[ref1] Patti ALA : *Why Vietnam.* Da Nang: Da Nang Publishing House;2008.

[ref2] Phong BD : President Ho Chi Minh - The one who laid the foundation for Vietnam’s integration with the world. *Communist Online Magazine.* 2021. 2734-9071. posted on July 31. Reference Source

[ref5] Quy DD : *Diplomacy before the National Resistance War and lessons on foreign affairs today.* People’s Army Newspaper online;2016. posted on December 15. Reference Source

[ref17] Selivanov IN : A New Collective Monograph of Russian and Vietnamese Scholars on Ho Chi Minh and his Political Legacy. *The Russian Journal of Vietnamese Studies.* 2021;5(1):182–190. 10.24411/2618-9453-2021-10009

[ref18] Tonnesson S : *Vietnam 1946 – How the War Began.* Hanoi: National Political Publishing House;2013.

