# Vietnam's foreign policy (1945-1946): Proactive in a fragile independence

**DOI:** 10.12688/f1000research.166625.3

**Published:** 2026-02-25

**Authors:** Quoc Dung Mai

**Affiliations:** 1 Ho Chi Minh City University of Industry and Trade, Ho Chi Minh, Vietnam

**Keywords:** Foreign policy, Proactive, Independence, Fragile;, 1945-1946

## Abstract

**Background:**

The August Revolution of 1945 established the Democratic Republic of Vietnam, but the nascent government immediately faced immense challenges from external forces like French colonialists and Chiang Kai-shek’s army, alongside internal difficulties. In this precarious situation, diplomacy emerged as a crucial strategic tool for the Vietnamese revolution during the 1945-1946 period, demonstrating a skillful blend of struggle and negotiation to safeguard independence.

**Methods:**

This study is grounded in the principles of dialectical and historical materialism, consistent with the viewpoint of the Communist Party of Vietnam. The main methods include: the historical method (systematic examination of events and policies), the logical method (reconstructing diplomatic strategies), intertextual analysis (comparing Party directives with diplomatic actions), critical discourse analysis (Ho Chi Minh’s statements), and comparative assessment (with other decolonization movements). Data were collected from declassified archival materials, legal texts, diplomatic records, and contemporary press.

**Results:**

During 1945-1946, Vietnam implemented an independent, self-reliant, and open foreign policy based on principles of equality and mutual assistance, with the core objective of protecting independence, sovereignty, and territorial integrity. This policy demonstrated strategic flexibility by conciliating Chiang Kai-shek’s forces to free up resources against the French, and by signing the Preliminary Accord and Provisional Agreement with France to gain time for resistance preparation. Vietnamese diplomacy also proactively established friendly relations with neighboring countries and major powers, sought international recognition, and committed to multilateral cooperation, thereby strengthening the legitimacy of the revolutionary government.

**Conclusion:**

In an extremely challenging situation, foreign affairs activities, under the leadership of the Party and President Ho Chi Minh, successfully protected Vietnam’s independence and enhanced the Democratic Republic of Vietnam’s prestige. The strategic lessons on foreign policy thinking from 1945-1946 have become a firm foundation for Vietnam’s modern foreign policy. They emphasize the harmonious combination of national independence, socialism, genuine patriotism, and internationalism, striving towards the ultimate goal of building a “prosperous people, strong country, democracy, justice, and civilization” in Vietnam.

## Introduction

The collapse of the Japanese Empire in August–September 1945 created a power vacuum in Southeast Asia. In Vietnam, the August Revolution culminated in the Declaration of Independence on September 2, 1945 a decisive yet exceedingly fragile act. The famine of 1944–1945 had left society exhausted; the state apparatus remained embryonic; and the reassertion of French authority, coupled with the presence of Chinese Nationalist forces in the North, generated a highly challenging external environment ( Marr, 1995; Goscha, 2016). Dinh et al. also affirm that the nascent revolutionary government was immediately confronted with a perilous situation, facing simultaneous pressures from multiple directions: in the North, more than 200,000 Chinese Nationalist troops entered to disarm the Japanese forces and intervened deeply in Vietnam’s internal affairs; in the South, British forces helped the French restore the colonial regime, triggering serious armed conflicts from September 1945 onward. Amid economic exhaustion and the severe consequences of the 1945 famine, Vietnam was compelled to prioritize social stabilization, consolidation of governmental authority, and the defense of the revolutionary achievements ( Dinh et al., 2008, p. 65).

Following the August Revolution, Archimedes L.A. Patti observed that “Hanoi alone became something like a center of underground and mysterious international movements,” with journalists and agents from “the U.S., Britain, France, China, the Netherlands, India, and the Soviet Union” converging on the city ( Patti, 2008, p. 577). Despite divergent agendas, imperialist powers and foreign reactionaries shared a singular objective: the dismantling of Vietnam’s nascent revolutionary government and the reversal of the achievements of the August Revolution.

Against this backdrop, diplomacy emerged as a vital instrument of the nascent regime: not only to secure international recognition, but also to buy time, consolidate domestic institutions, and mitigate the risk of eradication. Ho Chi Minh and the leadership of the Democratic Republic of Vietnam (DRV) pursued a series of balancing diplomatic initiatives: sending appeals (for instance, Ho’s letters to President Truman), negotiating with the French, making calculated concessions to the Chinese Nationalist forces in exchange for their withdrawal, while at the same time maintaining discreet contacts with the Soviet Union ( Patti, 2008; Tonnesson, 2013).

A matter of survival for Vietnam was the urgent need to affirm its national sovereignty and establish its legal status in the international arena. The search for a conciliatory solution to avoid a full-scale confrontation with France, while at the same time gaining time to consolidate forces and stabilize the country, led to President Ho Chi Minh’s policy of diplomatic negotiation. He underscored the necessity of upholding the principle of “dĩ bất biến ứng vạn biến” ( Ho Chi Minh National Academy of Politics, 2016, p. 197) that is, to use the unchanging to respond to the ever-changing, remaining steadfast in defending national independence while maintaining flexibility amid a complex and rapidly shifting international environment.

This paper examines the socio-political and cultural transformations in Vietnamese society that originate in 1945 and were rooted in early 19th-century dynamics. From the proclamation of independence and the founding of the Democratic Republic of Vietnam (DRV), the country has endured a tumultuous trajectory: two devastating wars, a national partition, and profound shifts in political and social structures. Across this period, new generations emerged, cultural values underwent adaptation (or rejection), and societal norms evolved a process that is still unfolding today. Notably, the Communist Party of Vietnam (CPV), established by Ho Chi Minh in the 1930s, persisted as the state’s unchanging leadership force. Since 1945, the CPV has functioned as the sole architect and guarantor of Vietnam’s most critical societal transformation ( Novakova O.V., 2015, p. 240-261). The diplomacy of the DRV constituted a blend of strategic initiative and adaptation to the constraints imposed by the international structure ( Walt, 1990; Wendt, 1992; Fairclough, 1992).

For smaller nations confronting imperial domination, total struggle encompassing diplomacy is imperative. Drawing on his global revolutionary experience, Ho Chi Minh, leader of Vietnam’s asymmetrical resistance, asserted: “Whoever seizes the diplomatic advantage will win” ( Ho, 2011a, p. 559). His flexible yet strategic foreign policy during 1945–1946 exemplifies this principle by securing critical leverage for the revolution.

Many Vietnamese studies have concentrated on the role of Ho Chi Minh and the Party in safeguarding independence through both military and diplomatic means. Luan (2001) characterizes the period 1945–1946 as a sequence of forced strategic choices, in which “diplomacy was the sharpest weapon” before armed forces became sufficiently strong. Phong (2021) emphasizes the dimension of national unity and how the DRV mobilized nationalist discourse to consolidate the nascent regime.

Marr (1995) reconstructs the domestic and international contexts of 1945; Patti (2008) provides U.S. perspectives through the experience of the OSS; Lawrence (2005) analyzes European and American viewpoints; Tonnesson (2013) argues that the outbreak of war in 1946 was not inevitable but rather the product of miscalculations, mutual suspicion, and colonial ambition. Goscha (2016) situates Vietnam within the broader regional transformations following World War II. Taken together, these studies illustrate the multidimensional nature of the period, in which local power, great-power rivalries, and international discourse all played significant roles.

From a theoretical perspective: Walt (1990) identifies strategies of small states as bandwagoning, balancing, or hedging; Jackson (1990) and Clapham (1996) examine the survival of “quasi-states” and weak polities. Constructivist and discourse-analytic approaches ( Wendt, 1992; Fairclough, 1992) explain how the DRV employed political language most notably citations from the Declaration of Independence to appeal to international normative frameworks, thereby seeking legitimacy. Skutnabb-Kangas (2000), though primarily concerned with linguistic and cultural rights, provides conceptual tools that highlight the importance of the “right to speak” and “right to be heard” in international relations—an idea highly relevant to the diplomatic struggles of a newly established state.

The historiography thus reveals several core themes in the scholarly discussion of Vietnam’s diplomacy during 1945–1946:

### Vietnam’s Civilized and Ethical Diplomacy: A Testament to Maturity and Progressive Values

Following the August Revolution, Vietnam faced multifaceted challenges, yet its foreign policy demonstrated remarkable civilizational maturity, compelling adversaries to acknowledge its legitimacy. As Dixee R. Bartholomew-Feis (2007, p. 357) notes, Vietnam’s diplomacy shattered colonial stereotypes, disproving the notion that the nation was “barbaric,” as French propaganda had long claimed. This sentiment was echoed by American journalist Panlo Hop, who, after visiting Vietnam in December 1945, affirmed that the Vietnamese people were a civilized nation deserving international recognition for their independence ( Ho, 2011b, p. 151).

The failure of France’s Instruction on December 10, 1946 which omitted any reference to its supposed “civilizing mission” further underscored Vietnam’s political and cultural autonomy ( Tonnesson, 2013, p. 296). This marked a decisive rejection of colonial rhetoric, positioning Vietnam as a democratic and progressive state fully capable of standing alongside other sovereign nations.

Ho Chi Minh, Vietnam’s revolutionary leader, articulated the nation’s diplomatic philosophy with clarity: “We must rely on strength. Strong strength and diplomacy win. The strength is like a gong, and diplomacy is its resonance. The louder the gong, the farther its sound carries” ( Ho, 2011c, p. 147).

This principle emphasized soft power as the cornerstone of Vietnam’s post-revolutionary diplomacy rooted in progressive cultural-ethical values and aligned with the nation’s historical and material conditions. From a dialectical-materialist perspective, Vietnam’s diplomacy was both a product and a driver of an advanced socio-political environment, one that upheld universally recognized legal and humanitarian principles.

Ho Chi Minh’s vision extended beyond resistance: he sought global solidarity for peace and shared prosperity. While condemning imperialist violations of the Atlantic Charter and the San Francisco Charter, he simultaneously advocated for Vietnam’s territorial integrity and peaceful coexistence ( Phong, 2021). This dual approach, steadfast in its sovereignty yet open to equitable international partnerships, is a prime example of Vietnam’s civilized and ethical statecraft, distinguishing it from the very forces that have denied Vietnam its right to self-determination.

### Vietnam’s Commitment to International Cooperation and Multilateral Engagement

Upon declaring independence, Vietnam proactively communicated its openness to global cooperation, addressing the French government, allied powers, and the United Nations with a clear message: Vietnam was a sovereign nation ready to collaborate in nation-building and global progress. As Ho Chi Minh articulated, “Any country including France that sincerely wishes to invest in Vietnam for mutual benefit will be warmly welcomed” ( Ho, 2011c, p. 145). He further emphasized Vietnam’s inclusive approach: “We will invite French experts, as well as American, Russian, or Chinese specialists, to assist in our national reconstruction” ( Ho, 2011b, p. 86).

Vietnam actively supported the creation of the Far East Advisory Committee, asserting its eligibility to appoint representatives and contribute to resolving regional challenges. This demonstrates Vietnam’s commitment to institutional diplomacy and its belief in collective problem solving. Ho Chi Minh’s visionary leadership transcended national liberation; his ideology, rooted in a progressive worldview, became not only a guiding doctrine for Vietnam’s Communist Party but also a significant contribution to global political thought ( Selivanov, 2021).

To secure international recognition, Vietnam’s diplomacy leveraged allied wartime commitments, particularly the principles of self-determination and equality among nations. President Ho Chi Minh engaged in high-level correspondence with leaders of the U.S., the UK, the Soviet Union, China, and the UN General Assembly, formally announcing Vietnam’s independence and seeking support.

Recognizing the ambiguous U.S. stance on Indochina, Vietnam strategically cultivated ties with American representatives including the U.S. Mission in Indochina and the Office of Strategic Services (OSS) to counterbalance French colonialists and Chiang Kai-shek’s forces. These efforts persuaded the U.S. to adopt a neutral position, mitigate external pressures and buy Vietnam’s critical time to consolidate its governance ( Quy, 2016).

Vietnam’s cultural diplomacy following the August Revolution (1945) yielded significant outcomes, projecting the nation’s identity globally, reinforcing broader foreign policy efforts, and establishing a crucial foundation for the subsequent resistance struggle ( Tung, 2018). This period marked a transformative phase in which Vietnam’s diplomatic strategies were deeply intertwined with its revolutionary ethos and the quest for international legitimacy.

This article examines the formation and evolution of Ho Chi Minh’s worldview, shaped over decades of revolutionary activism and the protracted struggle for Vietnamese independence. As Ngoc and Shpkovskaya (2022, p. 281-300) highlight, his ideological framework forged through global exposure (Europe, Asia, and America) and anti-colonial resistance became the bedrock of Vietnam’s foreign policy.

### While prior research has outlined the general characteristics of Vietnam’s 1945–1946 foreign policy, critical aspects remain underexplored

Although numerous studies have been conducted, a significant gap remains with regard to an integrated analysis that simultaneously meets three criteria: (i) an in-depth combination of domestic and international historical sources; (ii) a close linkage between discourse analysis and strategic analysis through the progression of diplomatic initiatives; and (iii) the placement of Vietnam within a comparative framework alongside other postcolonial states in order to highlight its distinctive characteristics. To fill this gap, the research focuses on addressing the following key research problems:

*The coherence between short-term and long-term objectives:* clarifying whether Vietnam’s primary priority was to secure immediate legal recognition or to establish durable strategic alignments for a protracted resistance;

*The relationship between core principles and diplomatic practice:* analyzing how the values of national independence, tactical flexibility, and anti-colonial solidarity were translated into specific diplomatic strategies;

*The distinctiveness of strategic action:* identifying the essential differences in Vietnam’s approach compared with other models liberation in the postcolonial context of national.

By addressing these issues, the article offers a systematic interpretation of early Vietnamese diplomacy, thereby elucidating the consistency of its strategic thinking and the profound historical significance of this formative period.

## Methods and data

### Research method

This study employs the historical and logical methods as its principal methodological foundations, in combination with discourse analysis and comparative historical approaches. Such an approach goes beyond the mere systematization of events, seeking instead to decode the strategic calculations and political meanings embedded within those developments.

Discourse analysis: Applying Fairclough’s method of three levels: (1) text, (2) discursive practice, (3) social practice. The objective: to explore how the DRV used terms such as “independence,” “equality,” and “peace” to connect with the international normative framework, thereby creating legitimacy.

Historical comparison: Comparing the behavior of the DRV with Indonesia (1945–1949) and India (1947) in order to clarify similarities/differences in “survival diplomacy.”

### Sources of data

Primary sources: (1) Political-legal documents: Declaration of Independence (2 September 1945); Ho Chi Minh’s letters to President Truman (1945–1946); Preliminary Agreement (6 March 1946); Provisional Agreement (14 September 1946). (2) Contemporary press: domestic newspapers such as Cứu Quốc, Nhân Dân; international newspapers and journals reflecting global perceptions of Vietnamese diplomacy. (3) Diplomatic and intelligence documents: telegrams, memoranda, and memoirs of relevant figures (for example: Archimedes L. A. Patti, OSS).

Secondary sources: (1) Research works of domestic and foreign scholars, typically David Marr, Christopher Goscha, Stein Tønnesson, Mark Lawrence, Nguyễn Phúc Lân, Bùi Đình Phong, etc. (2) Collections of documents and published materials: Documents of the Communist Party of Vietnam, Central Committee resolutions, Politburo directives, reports of Party Congresses on foreign policy.

Some limitations in the research process: The author’s access to some archives is still limited; some sources are assessments of Vietnamese and international authors which may still carry ideology; some memoirs and recollections (used in a limited way in this article) may be subjective. Therefore, some issues in this study may need further discussion and research.

## Results and Discussion

### Foreign policy in the years 1945-1946

The success of the August Revolution in 1945 led to the establishment of the Democratic Republic of Vietnam (DRV). In the Declaration of Independence on September 2, 1945, the stance of the representative of the DRV was an important discursive opening move: Ho Chi Minh directly cited the U.S. Declaration of Independence and the French Declaration of the Rights of Man and of the Citizen to lay the foundation of international legitimacy for a new state. The famous sentence: “All men are created equal. They are endowed by their Creator with certain unalienable rights; among these are life, liberty, and the pursuit of happiness.” ( Ho, 2000a, p. 1). This was not merely a cultural quotation but a discursive strategy: linking the national aspiration with universal norms. The DRV used language to assert subjectivity: citing international declarations and making universal claims to attract sympathy (legitimacy by resemblance). According to Wendt (1992), “anarchy is what states make of it”; the DRV sought to shape part of the meaning of the international arena by embedding itself into the grand narrative of national self-determination and human rights.

Following that, the Declaration affirms: “The Provisional Government of Vietnam, representing the entire Vietnamese people, declares complete separation from colonial relations with France, annulling all treaties France imposed on Vietnam and abolishing all French privileges in Vietnamese territory” ( Ho, 2000a, p. 3). The document further affirmed: “Vietnam has the right to enjoy freedom and independence, and has in fact become a free and independent nation. The Vietnamese people are determined to mobilize all their spiritual and material resources their lives and property to safeguard these fundamental rights” ( Ho, 2000a, p. 4). This historic declaration not only marked Vietnam’s independence but also established the principle that only sovereign nations possess the right to determine their own foreign policies. The study shows that the initial objectives of the DRV were: (i) to assert its political existence before the international community; (ii) to seek humanitarian and political support; (iii) to delay colonial restoration through a political–diplomatic strategy.

The nascent government faced an extremely precarious situation: Vietnam’s independence lacked international recognition; over 30,000 allied troops (including French colonial forces and Chiang Kai-shek’s army) were stationed in the country, with both groups actively working to overthrow the revolutionary government; Viet Minh possessed only about 80,000 poorly equipped troops; opposition parties (Việt Quốc and Việt Cách), backed by foreign powers, controlled half of the Provisional Government’s ministries.

The nation confronted severe challenges: an empty national treasury, the lingering effects of the 1945 famine that claimed over two million lives, a 90% illiteracy rate, and extremely low agricultural productivity (about 12 quintals of rice/ha).

These overwhelming difficulties threatened the collapse of the fledgling government. Using any conventional measure of material strength, the Democratic Republic of Vietnam was destined for rapid collapse. However, through strategic methods of struggle including innovative diplomatic efforts Vietnam gradually overcame these challenges, laying the groundwork for the subsequent resistance war against the French colonial forces.

*Regarding foreign affairs,* under the new conditions of direct leadership of the government, the Party outlined internal and external policies to serve as the cause of resistance war and national construction to victory. Foreign affairs are placed in an important position with a system of viewpoints, strategies, and tactics on Vietnam’s relations with the world.

On October 3, 1945, the Provisional Government of the Democratic Republic of Vietnam issued a “Communication on the foreign policy of the Democratic Republic of Vietnam”, which clearly stated that Vietnam’s foreign policy was built on the basis of Vietnamese practice and the international situation. This means that the Vietnamese people themselves draw up an independent foreign policy and direction on the basis of the requirements and tasks of the Vietnamese revolution, but at the same time must be in line with international standards, appropriate corresponding to the trend of the times.

Vietnam’s foreign policy goal is to contribute to “bringing the country to complete and permanent independence”. It is a consistent affirmation of the foreign policy mission to ensure the interests of the nation and the nation, and to ensure basic national rights such as national independence, national sovereignty, territorial integrity, and unity.

The communiqué mentioned Vietnam’s foreign policy with a number of key subjects in international relations such as: major countries, countries in the anti-fascist allies, “Vietnam is extremely friendly and sincerely cooperates with on the stance of equality and mutual love”; “particularly for the French government, which advocates domination of Vietnam, it resolutely opposes it”; with neighboring countries, the communiqué emphasizes friendship, cooperation and equality; With the two countries of Cambodia and Ai Lao, Vietnam advocates that “the line of communication with the nation’s self-determination as the foundation must be even closer”…

*Regarding foreign policy principles,* Vietnam’s diplomacy takes the principles of the Atlantic Charter as the foundation, and the directive of the Central Executive Committee on the National Resistance War dated November 25, 1945 stated: “persist with the diplomatic relations with other countries on the principle of equality and mutual assistance. Particular attention must be paid to these: one is that diplomacy is to make one’s country fewer enemies and more allies than most; second, for diplomacy to be successful, we must show our strength” ( Communist Party of Vietnam, 2000b, p. 27).

Upholding the goals and principles, and at the same time willingness to implement an open foreign policy is a unique feature of Vietnam’s new foreign policy. In his Call to the United Nations, Ho Chi Minh clearly stated the foreign policy of the Democratic Republic of Vietnam: “In its foreign policy, Vietnamese people adhere to the following principles: 1. As for Laos and Myanmar, Vietnam respects the independence of these two countries and expresses its desire to cooperate on the basis of absolute equality between sovereign countries: 2. For democratic countries, Vietnam is ready to implement an open-door policy and cooperate in all fields: a) Vietnam won a favorable reception for investment from capitalists and engineers in all its industries. b) Vietnam is ready to expand its ports, airports and roads for international trade and transit. c) Vietnam accepts joining all international economic cooperation organizations under the leadership of the United Nations. d) Vietnam is ready to conclude with the navy and army forces within the framework of the United Nations special security agreements and treaties relating to the use of some naval bases and airspace ( Ho, 2000a, p. 469-470).

*Regarding the foreign policy motto,* Vietnam’s diplomacy has thoroughly grasped the viewpoints of independence, self-reliance, and self-reliance. In international relations, it is necessary to grasp the motto of being persistent in principle, firm in strategy, but flexible and flexible in strategy: “Our unchanging goal is still peace, unity, and independence establishment, democracy. Our principles must be firm, but our strategies must be flexible” ( Ho, 2000b, p. 319).

The Party’s foreign policy motto shows its proactive, positive and self-reliant stance; releases me by my strength; “If we are strong, then they will care about us. If we are weak, we are just an instrument in the hands of others, even if that person can be our ally” ( Communist Party of Vietnam, 2000a, p. 244).

### The results of the implementation of the policy

In fact, under the agreements reached by the Allied powers at the Potsdam Conference, the area north of the 16th parallel was assigned to the Chinese Nationalist forces of Chiang Kai-shek for the disarmament of Japanese troops, while the area south of the 16th parallel was entrusted to British forces. In practice, the British facilitated the return of French colonial forces to reoccupy Cochinchina from September 1945. By late 1945 and early 1946, France sought to re-establish its colonial apparatus through military means, while Chinese Nationalist troops in the North not only disarmed the Japanese but also exerted considerable political and economic pressure on the government of Ho Chi Minh. This situation placed Vietnam at risk of partition or a renewed loss of independence. The urgent task was to “maintain the revolutionary government” and to “avoid military confrontation while forces remained unequal”; at this juncture, it was essential to calculate carefully in light of practical conditions, for “the issue is to know oneself and know the other, to objectively assess domestic and international conditions of advantage and disadvantage in order to adopt the correct policy” ( Communist Party of Vietnam, 2000b, pp. 43-44). Consequently, seeking a diplomatic solution to reduce external pressures while gaining time to consolidate internal strength became a strategic priority.

Vietnam’s new foreign policy has been effective since the beginning of its revolutionary government. In the years 1945-1946, the Vietnamese revolution had to deal with many dangerous enemies. On the basis of correctly determining “Our main enemy at this time is the invading French colonialists, must focus the fire of struggle on them, the Party has implemented a clever foreign policy: at times advocated “friendly Sino-Vietnamese”, détente with Chiang Kai-shek to limit their actions, so that they would not oppose the Vietnamese revolution, but leave their hands free to deal with the French colonialists, sometimes making peace with the French to push Chiang’s troops back home, implementing the policy of “reconciling peace and harmony” to achieve the goal”. These are examples of flexibility in strategy and ingenuity in taking advantage of conflicts between hostile forces to bring the Vietnamese revolution into a dangerous situation.

The presence of approximately 200,000 Kuomintang (KMT) troops in northern Vietnam posed an immediate challenge. The DRV, in order to avoid direct confrontation with the still-powerful KMT forces and to prevent them from supporting the French, accepted certain concessions (providing food supplies, granting economic privileges to China). Scholars have described this as a form of “necessary concession” ( Chen, 1996). From a strategic perspective, this constituted a risk-avoidance behavior: conceding partially in exchange for troop withdrawal and avoiding immediate annihilation.

In this context, the French government also pursued its own calculations. Postwar France was striving to restore its colonial position, yet its military capabilities were constrained. At the same time, the presence of more than 200,000 Chinese Nationalist troops in northern Vietnam created significant pressure on the French, compelling Paris to negotiate with Vietnam in order to replace these forces, to “negotiate with China to persuade them to withdraw their troops quickly from northern Indochina and allow French forces to return” ( Devillers, 2003, p. 122). This convergence of interests created an opening for diplomatic engagement between Vietnam and France.

The contacts between Jean Sainteny and Vietnamese representatives from late 1945 marked the opening move of a tense diplomatic contest aimed at stabilizing a situation on the brink of war. For Vietnam, conciliation did not signify unconditional compromise but rather a material calculation: the use of diplomacy to compensate for limitations in military capacity.

Throughout four months of negotiations (December 1945–March 1946), President Ho Chi Minh skillfully exploited divisions within the French leadership between Sainteny’s inclination toward dialogue and the hawkish stance of Georges Thierry d’Argenlieu. Vietnam’s persistent demand for legal recognition as an independent state, possessing its own government and army, generated countervailing pressure on France. Ultimately, in pursuit of the strategic objective of replacing Chinese Nationalist forces in northern Vietnam, France was compelled to accept core concessions. The Preliminary Agreement of 6 March 1946 was the outcome of this sustained struggle, enabling Vietnam to gain valuable time to prepare for the protracted resistance that would follow.

The fact that President Ho Chi Minh on behalf of the government of the Democratic Republic of Vietnam signed with the representative of the French Government the Preliminary Agreement on March 6, 1946 and the Provisional Agreement on September 14, 1946, the optimal foreign policy solution to protect the achievements of the revolution, taking advantage of more time to prepare for the long-term resistance war against the French, which our Party knows is inevitable.

The March 6, 1946 Agreement was a significant step: France recognized the DRV as a “free state” within the French Union; in return, on the ground, France was granted the right to bring troops back into northern Vietnam in order to resolve the state of disorder. Tonnesson (2013) described this as a “truce game” the DRV obtained conditional recognition in exchange for preventing an immediate military reconquest. Although full independence was not achieved, Vietnam compelled France to acknowledge its existence as a state possessing its own institutions. This recognition enhanced the international legitimacy of the Democratic Republic of Vietnam and provided an important foundation for subsequent diplomatic activities. Vietnam used the Agreement as a strategic platform to continue its struggle at the Fontainebleau Conference and in other international engagements. The supplementary agreement signed concurrently with the Preliminary Agreement of 6 March 1946, witnessed by representatives of the British, American, and Chinese Nationalist delegations, compelled the Chinese Nationalist forces to withdraw promptly from northern Vietnam, to be replaced by 15,000 French troops. French forces were to evacuate Vietnam within five years, withdrawing one-fifth each year. With the departure of the Chinese Nationalists, “the Viet Cach and Viet Quoc, having lost their backing, hastily followed them out. Our acceptance of membership in the Indochinese Federation within the French Union also deprived the British and Americans of any pretext to remain and carve up Vietnam. Thus, with a single move, we removed four adversaries at once: the Chinese Nationalists, the Americans, the British, and their local proxies” ( Ho Chi Minh National Academy of Politics, 2001, p. 128).

In July 1946, the Franco - Vietnamese delegation met at Fontainebleau; the negotiations ended without reaching a comprehensive agreement. Ho Chi Minh returned to France in September 1946 to continue talks; the September 14, 1946 Provisional Agreement laid out a series of economic arrangements and commitments to further negotiation, but it failed to resolve the fundamental conflict over ultimate sovereignty. Mutual trust had already eroded.

The challenge for the DRV was how to survive among great powers. The pragmatic strategy included: exploiting contradictions among major powers, making partial concessions (to China), signing a temporary settlement with France in exchange for the withdrawal of the Kuomintang forces, and simultaneously seeking recognition from both the United States and the Soviet Union (though not immediately successful). This illustrates the concepts of hedging and balancing in IR ( Walt, 1990).

Regarding Southeast Asian countries. For Laos and Cambodia, the policy of the Communist Party of Vietnam is: “Unifying the Vietnam - Cambodia - Laos front against aggression”. On October 30, 1945, the Agreement on Military Alliance between the Government of Itxata and the Government of the Democratic Republic of Vietnam along with the Agreement on the Alliance of Laos, Vietnam was signed and began to be implemented. For Asian countries, Vietnam actively opens friendly relationships. Immediately after the birth of the Democratic Republic of Vietnam, the Government sent a representative to Bangkok (Thailand) to enlist the support of the government and the people of the country. The Vietnamese Government’s special envoy held meetings with diplomatic representatives of India and Indonesia, creating the basis for Vietnam’s relations with these countries.

In fact, immediately after winning power and establishing a new Vietnamese State, the Party was rightly aware of the position and role of foreign affairs in the resistance war and national construction. On that basis, the Party soon established an independent and self-reliant foreign policy, including the following: foreign affairs objectives and tasks, arrangement of forces, determination of principles, mottos and methods of diplomatic struggle of the Vietnamese revolution. The new Vietnamese state’s foreign policy “renovated the relationship between the country and its colony and its neighbors near and far - including relations with major countries, opening a new history in international relations” ( Luan 2001, p. 82).

Before and after independence, as the head of the Provisional Government, President Ho Chi Minh sent many emails and letters to the heads of state and foreign ministers of countries and organizations such as the United States, China, the Soviet Union and the United Nations. The letters and telegrams show Vietnam’s diplomatic views; that is, from the beginning, it has tried to expand relations with other countries, especially great powers, to enlist the recognition of Vietnam’s legal status. The independent South, thereby establishing a host position in communication with foreign countries, protects the newly established democratic republic. However, in the complicated context of the world situation at that time, the Vietnamese people had to fight against French colonialists and protect the revolutionary achievements in an almost lonely situation. However, while the fledgling revolutionary government was in a situation of “thousands of pounds hanging by a hair,” enemies inside and outside, facing difficulties on all sides, Vietnam’s foreign affairs had successfully completed its tasks, contributing to protecting and strengthening the revolutionary government, preparing forces for a long resistance war, and leaving valuable lessons for foreign affairs in the current period.

The effect of diplomacy in seeking consensus and attracting international public opinion was constrained by structural limits (the strategic priorities of the great powers), which ultimately determined the outcome. The United States, prioritizing Europe, could not sacrifice French interests; the Soviet Union was not yet actively engaged in Southeast Asia. Both factors meant that the DRV could not rely on external guarantees to prevent war ( Lawrence, 2005). Nevertheless, the negotiation period of 1945–1946 was not in vain: the DRV gained time to organize, recruit self-defense forces, and build an internal political foundation ( Marr, 1995; Patti, 2008). The period 1945-1946 was the most special and meaningful period in the nation’s history, and this was also a memorable period of Vietnamese diplomacy. With the correct, flexible and resolute policies and measures of President Ho Chi Minh, he created opportunities to take advantage of them to win.

Comparing the case of Vietnam with Indonesia, Reid (1974) refers to the Southeast Asian context after World War II, in which Vietnam’s declaration of independence on September 2, 1945, is mentioned as part of the wider wave of anti-colonial revolutions sweeping across Asia. Reid argues that Vietnam (under Viet Minh leadership) and Indonesia both exploited the “power vacuum” following Japan’s surrender to proclaim independence. However, Vietnam faced direct military intervention from France (with British support in southern Vietnam), while Indonesia confronted the Dutch, though in a context where the British were also temporarily present (1945–1946). Reid notes that Vietnam’s early entanglement in the Indochina War (from 1946) shaped international perceptions of Indonesia’s independence movement differently, placing greater pressure on the Netherlands to negotiate. Unlike Indonesia, which benefited from its archipelagic geography, Vietnam occupied a highly sensitive geopolitical position, a “crossroads” of continental power currents. At this juncture, Vietnamese diplomacy demonstrated remarkable acuity: it not only seized the “power vacuum” to take control of the state apparatus, but also proactively constructed a “space for survival” through the signing of the Preliminary Agreement of 6 March 1946. This constituted a pivotal diplomatic decision, enabling Vietnam to divide its adversaries, secure the withdrawal of 200,000 Chinese Nationalist troops, and concentrate its efforts on confronting its principal enemy - French colonialism.

Guha (2008), while not focusing extensively on Vietnam, does refer to it in the international context, mentioning the Cold War in Asia (the Korean War, the Vietnam War) to compare the instability of other Asian states with India. Guha points out that while many Asian countries (such as China, Pakistan, Vietnam) experienced war, instability, or authoritarian regimes, India maintained a continuous parliamentary democracy. Guha also highlights countries that gained independence after 1945 (Indonesia, Ghana, Vietnam, etc.) to stress India’s distinctiveness being both vast in scale and able to preserve a multi-party democracy. However, the “instability” to which Ranajit Guha refers in the Vietnamese case should be understood as a process of forging diplomatic resilience amid the encirclement of “internal enemies and external aggressors”. Whereas India benefited from a structured transfer of power by Britain, the government of the Democratic Republic of Vietnam had to rely on its own efforts to assert its legitimacy. The organization of the first General Election in January 1946 and the establishment of the Government of National Resistance and Coalition represented exceptionally astute internal diplomatic measures, designed to demonstrate to the international community that Vietnam possessed not only military strength but also a constitutional and democratic institutional framework, thereby securing a more equal footing at the negotiating table with France.

Regarding diplomacy, Reid (1974) and Guha (2008) argue that postcolonial states often employed a mixture of negotiation and military struggle; Vietnam’s key difference lay in the simultaneous presence of multiple great powers on its territory (China, France) and in France’s determination to restore its empire. The distinctive hallmark of Vietnamese diplomacy lay in the strategy of “peace in order to advance” a skillful synthesis of firm principles regarding independence and flexible tactical methods. Encircled by major powers, Vietnam elevated diplomacy to a strategic front equal to the military sphere. The signing of the Preliminary Agreement of 6 March 1946 and subsequently the Modus Vivendi of 14 September 1946 was not merely an act of concession, but rather an art of “buying time” to consolidate forces. This demonstrated the early contours of a multilateral diplomatic outlook: from as early as 1945, Vietnam sought to internationalize the Indochina question, attempting to engage the United States and international organizations in pursuit of recognition, rather than confining itself solely to bilateral relations with the colonial power.

The conflict escalated in northern Vietnam; the November 1946 Haiphong incident (when French forces shelled the city) marked the climax. Official diplomacy collapsed, and December 1946 signaled the beginning of the full-scale resistance war against France. That diplomatic strategy left behind extremely valuable lessons, not only meaningful during the years of fighting the French and the Americans but also meaningful today.

## Some lessons learned

### First, the lesson emphasizes the legitimacy and strength of the revolutionary government

In August 1945, when the Second World War ended, the global situation changed at an extremely rapid pace. Thanks to correctly predicting the world and domestic situation, knowing that Japan was about to surrender to the Allies and that the Japanese army in Vietnam was extremely confused, the Indochina Communist Party decided to seize the opportunity and launch a General Uprising to seize power. The Party advocates that it must gain power and declare independence before the allied troops enter, promote the position of the Democratic Republic of Vietnam, gain legal status for the new government, and take advantage of international recognition to facilitate transactions with allies.

To create a legal basis and official name for the new government, the Revolutionary Command, which had just returned to Hanoi, decided to reform the National Liberation Committee of Vietnam into the Provisional Government of the Democratic Republic of Vietnam. On August 28, 2945, some units of the Republic of China’s army began to move into North Vietnam, and an independence declaration ceremony was held before Chiang’s army arrived in Hanoi. On September 2, 1945, President Ho Chi Minh read the Declaration of Independence on the nation and the world, affirming that Vietnam has the right to enjoy freedom and independence and has truly become a free and independent country…. the entire Vietnamese people are determined to use all their spirit and force, their lives and property, to maintain that right to freedom and independence…

After declaring independence on September 3, 1945, the government held its first session, proposing six major tasks, including organizing the National Assembly election as soon as possible throughout the country. On January 6, 1946, the first General Election in Vietnam’s history was held successfully. On March 6, 1946, Vietnam signed a Preliminary Agreement with France. Thus, in just a short time, thanks to the strength of solidarity of the entire nation, Vietnam established a completely legal and constitutional government, representing all Vietnamese people to perform their functions. President Ho Chi Minh also invited former Emperor Bao Dai to join the new government as a Government Advisor, continuing to add to the government apparatus many former Ministers of the Nguyen Dynasty; therefore, Vietnam the new South wants to tell the world that the key elements of the old regime all recognize and cooperate with the new regime.

On October 3, 1945, one month after declaring independence, the Ministry of Foreign Affairs of the Provisional Government issued a communiqué on foreign policy of the Democratic Republic of Vietnam, affirming the goal of striving for complete independence. The Communiqué was the State’s first document on foreign affairs, orienting the Party’s foreign affairs activities during the resistance war to build the nation, but first of all, to take advantage of and create momentum with allied forces in Vietnam. In the last few months of 1945 and early 1946, President Ho Chi Minh also sent many letters and diplomatic notes to major countries such as the US, UK, Soviet Union, China and the President of the United Nations General Assembly announcing life and affirming the legitimacy of the Democratic Republic of Vietnam, denouncing France’s return to invade Indochina.

It can be said that the above-mentioned diplomatic strategies have contributed to enhancing the legitimacy and strength of the revolutionary government to confront aggressive forces, create the ability to add friends and reduce enemies, and create favorable conditions for foreign countries. Activities of the young revolutionary government.

### Second, the lesson on how to distinguish enemies

After the success of the August Revolution, a serious challenge for the Vietnamese revolution was simultaneously dealing with many opposing military forces in major countries present in Vietnam at the same time.

The skillful and correct strategies mentioned above are a novel step in Vietnam, appeasing the opposition of the Republic of China and Vietnam, contributing to the prevention of many sabotage and subversion plots of the enemy but still ensuring the principle of maintaining a strong government in Vietnam.

It can be said that the distinction between “friends and enemies” in the previous period was the basis for the Vietnamese Party to form the viewpoint of “partner and object” in the period of international integration.

### Third, the lesson of knowing how to make concessions at the right time, make concessions with limits, and make concessions with principles

On September 2, 1945, the Democratic Republic of Vietnam was born and faced many difficulties in economics, politics, culture, security, and defense. Faced with this situation, in terms of diplomacy, the Party and the Government have implemented a policy of temporary concessions and peace while still ensuring the principles of national independence and sovereignty.

In the current period, the world and regional situation have undergone many unpredictable changes, making Vietnam’s defense and homeland protection, especially maritime security, a difficult and challenging task. From the historical lessons mentioned above, the Party and the State of Vietnam have inherited and creatively applied them to foreign affairs activities during the new period.

## Conclusion

The 1945–1946 period illustrates a diplomacy that was both proactive and constrained by objective limitations. The Government of the Democratic Republic of Vietnam (DRV) leveraged international discourse, sought to balance among partners, and made strategic concessions to prolong its survival. Although war ultimately broke out, the years 1945–1946 cannot be regarded as a pure failure; rather, they constituted a phase of learning, cadre training, and the initial shaping of fundamental principles of foreign policy, most notably the idea of “multilateralization and diversification” an orientation that continues to exert long-term influence on Vietnam’s foreign policy.

Theoretically, the Vietnamese case demonstrates that even small states can exercise agency, despite being heavily constrained by the international power structure. Practically, the lesson for today’s vulnerable states is the need to flexibly combine legal-normative discourse with pragmatic diplomatic strategy, thereby expanding policy space and safeguarding national interests.

After the August Revolution of 1945, the country faced enormous difficulties that seemed insurmountable. However, the DRV’s external activities opened up a new front of struggle, contributing to the defense of national independence while simultaneously affirming the DRV’s prestige and resilience in the international arena.

The strategic lessons of foreign policy thinking from the 1945–1946 period became the foundation for Vietnam’s contemporary external orientation. National interests must be situated in harmony with socialism, combining genuine patriotism with internationalism, aiming toward the highest goal of building a country that is “prosperous, strong, democratic, equitable, and civilized.” This has provided the basis for the formation, development, and refinement of Vietnam’s foreign policy of independence, autonomy, openness, and the multilateralization and diversification of international relations in the modern era.

## Ethical considerations

No ethical approval or consent was required.

## Data Availability

The data used in this study were obtained from secondary sources. Raw data were not collected and used in this particular article.

## References

[ref6] Bartholomew-Feis DR : *OSS and Ho Chi Minh – Surprise allies in the war against Japanese fascists.* Hanoi: World Publishing House;2007.

[ref19] Chen J : *China’s road to the Korean War: The making of the Sino–American confrontation.* Vol.52; New York: Columbia University Press;1996; p.534. 10.2307/40203228

[ref20] Clapham C : *Africa and the international system: The politics of state survival.* Cambridge: Cambridge University Press;1996.

[ref3] Communist Party of Vietnam: *The complete Party document.* Vol.7. Hanoi: National Political Publishing House;2000a.

[ref4] Communist Party of Vietnam: *The complete Party document.* Vol.8. Hanoi: National Political Publishing House;2000b.

[ref31] Devillers P : *Paris Saigon Hanoi*. (Hoang HD, trans.). Ho Chi Minh City: Ho Chi Minh City General Publishing House;2003.

[ref32] Dinh XL : *Selected topics on the revolutionary line of the Communist Party of Vietnam.* Hanoi: Political Theory Publishing House;2008.

[ref30] Fairclough N : *Discourse and social change.* Cambridge: Polity Press;1992.

[ref21] Goscha C : *Vietnam: A new history.* New York: Basic Books;2016.

[ref22] Guha R : *India after Gandhi: The history of the world’s largest democracy.* London: Macmillan;2008.

[ref9] Ho CM : *Complete volume.* Vol.3. Hanoi: National Political Publishing House;2011a.

[ref7] Ho CM : *Complete volume.* Vol.4. Hanoi: National Political Publishing House;2000a.

[ref10] Ho CM : *Complete volume.* Vol.4. Hanoi: National Political Publishing House;2011b.

[ref11] Ho CM : *Complete volume.* Vol.6. Hanoi: National Political Publishing House;2011c.

[ref8] Ho CM : *Complete volume.* Vol.7. Hanoi: National Political Publishing House;2000b.

[ref33] Ho Chi Minh National Academy of Politics : *Textbook of Political Secondary School: History of the Communist Party of Vietnam.* Hanoi: Internal circulation;2001.

[ref34] Ho Chi Minh National Academy of Politics : *Ho Chi Minh: A Chronological Biography.* Vol.3. Hanoi: National Political Publishing House;2016.

[ref23] Jackson R : *Quasi-states: Sovereignty, international relations and the Third World.* Cambridge: Cambridge University Press;1990.

[ref24] Lawrence MA : *Assuming the burden: Europe and the American commitment to war in Vietnam.* Berkeley: University of California Press;2005.

[ref25] Marr DG : *Vietnam 1945: The quest for power.* Berkeley: University of California Press;1995. 10.1525/9780520920392

[ref13] Ngoc NTB Shpakovskaya MA : Ho Chi Minh’s worldview and Vietnam’s foreign policy during the period of renewal. *Southeast Asia: Current Problems of Development.* 2022;4(4):281–300. 10.31696/2072-8271-2022-4-4-57-281-300

[ref14] Nguyen PL : *Modern Vietnamese diplomacy 1945–1946.* Hanoi: National Political Publishing House;2001.

[ref15] Nguyen TT : Ho Chi Minh with the construction of Vietnamese cultural diplomacy in 1945–1946. *Foreign Relations Journal – Central Committee for External Relations.* 2018;4(102). ISSN: 1859-2899.

[ref16] Novakova OV : Independent Vietnam: democracy versus traditionalism, 1945–1946 – the early XXI century. *The Russian Journal of Vietnamese Studies.* 2015;1(5):240–261.

[ref1] Patti AJ : *Why Vietnam? Prelude to America’s albatross.* Berkeley: University of California Press;2008.

[ref2] Phong BD : President Ho Chi Minh – The one who laid the foundation for Vietnam’s integration with the world. *Communist Online Magazine.* 2021. ISSN: 2734-9071. Published July 31. Reference Source

[ref5] Quy DD : *Diplomacy before the National Resistance War and lessons on foreign affairs today.* People’s Army Newspaper online.2016. Published December 15. Reference Source

[ref26] Reid A : *The Indonesian national revolution, 1945–1950.* London: Longman;1974.

[ref17] Selivanov IN : A new collective monograph of Russian and Vietnamese scholars on Ho Chi Minh and his political legacy. *The Russian Journal of Vietnamese Studies.* 2021;5(1):182–190. 10.24411/2618-9453-2021-10009

[ref29] Skutnabb-Kangas T : *Linguistic genocide in education – or worldwide diversity and human rights?* Mahwah, NJ: Lawrence Erlbaum;2000.

[ref18] Tonnesson S : *Vietnam 1946: How the war began.* Berkeley: University of California Press;2013.

[ref27] Walt SM : *The origins of alliances.* Ithaca, NY: Cornell University Press;1990.

[ref28] Wendt A : Anarchy is what states make of it: The social construction of power politics. *International Organization.* 1992;46(2):391–425. 10.1017/S0020818300027764

