# Supplementary material for: Vietnam's foreign policy (1945-1946): Proactive in a fragile independence
Source: F1000Res. 2026 Feb 25;14:643. Originally published 2025 Jul 3. [Version 3] doi: 10.12688/f1000research.166625.3 (PMC13107108; doi:10.12688/f1000research.166625.3)
Supplement: Peer review history

# Reviewer report: Reviewer response for version 3

**DOI:** 10.5256/f1000research.196586.r475953

**Published:** 2026-05-14

**Authors:** Mansur Juned

**Affiliations:** 1 Universitas Pembangunan Nasional Veteran Jakarta, Jakarta, Special Capital Region of Jakarta, Indonesia

This is a very interesting article, and personally, I expect a comprehensive historical analysis of Vietnam’s foreign policy at the end of World War 2. This article successfully highlights the acute geopolitical pressures confronting the nascent DRV, effectively mapping the intersecting imperial and regional forces (French, British, and Chinese Nationalist) that threatened the state's survival. It accurately identifies diplomacy as a vital, asymmetric tool used by Ho Chi Minh to buy time, fracture adversary coalitions, and consolidate domestic institutions before the outbreak of full-scale war.

 However, I perceive that several issues need to be addressed to improve the overall quality of the manuscript.

 First, the article treats the DRV as a monolithic actor, largely ignoring internal political fragmentation, grassroots revolutionary perspectives, or the intense domestic controversies that surrounded compromises like the March 6 Preliminary Agreement. Additionally, this study suffers from "archival silences," relying heavily on official elite documents while neglecting non-elite perspectives.

 Second, the author lists multiple methodologies—including historical reconstruction, intertextual analysis, and Fairclough’s Critical Discourse Analysis (CDA) —but applies them superficially. For instance, CDA fails to expose deeper ideological contestations or power dynamics, instead taking state discourse at face value to prove the effectiveness of the DRV’s “internationalist language.” Moreover, the internal power dynamics in Vietnam have not been fully explored. Furthermore, the comparative historical analysis juxtaposing Vietnam with Indonesia and India lacks the systemic depth required to illustrate the structural differences in their respective decolonization pathways and survival diplomacy.

 Furthermore, the methodology implemented in the research should be defined more clearly to allow method replication by others.

 Third, I perceive that the if the author wanted to dissect the international power play and how the DRV translated it into their novel perspective of foreign policy, it could be benefit by using the neoclassical realism perspective on how the DRV as agent translated the structure into their own action represented in their foreign policy.

 Fourth, The core historical claim—that tactical diplomatic concessions successfully secured breathing room for the revolutionary government—is valid and well supported by the timeline of events. However, the analytical leap in this conclusion is problematic. The author asserts that the ad hoc survival tactics of 1945–1946 directly laid the foundation for Vietnam's contemporary foreign policy of "multilateralization and diversification.” This long-term causal linkage is stated, rather than rigorously proven. Ultimately, the conclusion merely summarizes historical beats rather than synthesizing new scholarly insights or adequately answering the proposed research questions.

Is the work clearly and accurately presented and does it cite the current literature?

Partly

If applicable, is the statistical analysis and its interpretation appropriate?

Not applicable

Are all the source data underlying the results available to ensure full reproducibility?

Partly

Is the study design appropriate and is the work technically sound?

Partly

Are the conclusions drawn adequately supported by the results?

Partly

Are sufficient details of methods and analysis provided to allow replication by others?

No

Reviewer Expertise:

Geopolitics and Geoeconomics

I confirm that I have read this submission and believe that I have an appropriate level of expertise to confirm that it is of an acceptable scientific standard, however I have significant reservations, as outlined above.

# Reviewer report: Reviewer response for version 3

**DOI:** 10.5256/f1000research.196586.r467222

**Published:** 2026-04-23

**Authors:** Korwa Adar

**Affiliations:** 1 Institute of Diplomacy and International studies, University of Nairobi, Nairobi, Nairobi County, Kenya

The author clearly articulates Vietnam’s foreign policy from conceptual and theoretical perspectives. The author sets the stage first by examining the competing epistemology to isolate the gap in the literature which, thereafter, is methodologically analysed within the context of research problems. The paper, because of its originality, makes useful contribution in the ongoing debate on the Vietnam’s foreign policy.

 Methodology:

 Using historical and logical perspectives, particularly Fairclough’s three levels, as his central methodological analytical points for departure, the author has not only clearly demonstrated his understanding of the complexities inherent in the region in which Vietnam exerts her foreign policy interests but the author’s success in tracing Vietnam’s foreign policy behavioral patterns.The choice of period, the time-frame, in which Vietnam’s foreign policy is assessed, is useful particuarly because of the underlying and intricate internal and external structural systemic circumstances in which the country found itself.It is this latter scenario which makes the paper interesting and publishable.  

Is the work clearly and accurately presented and does it cite the current literature?

Yes

If applicable, is the statistical analysis and its interpretation appropriate?

Yes

Are all the source data underlying the results available to ensure full reproducibility?

Yes

Is the study design appropriate and is the work technically sound?

Yes

Are the conclusions drawn adequately supported by the results?

Yes

Are sufficient details of methods and analysis provided to allow replication by others?

Yes

Reviewer Expertise:

NA

I confirm that I have read this submission and believe that I have an appropriate level of expertise to confirm that it is of an acceptable scientific standard.

# Reviewer report: Reviewer response for version 3

**DOI:** 10.5256/f1000research.196586.r462045

**Published:** 2026-03-13

**Authors:** Ha Trieu Huy

**Affiliations:** 1 Vietnam National University, Ho Chi Minh City, Vietnam

Thank you for revising your article. Your piece now has stronger arguments on Ho's efforts to preserve his regime's political power and resist against the return of the French in the earliest chapter of the new Indochina after the Second World War. 

 However, I encourage you to make further minor revisions to improve this paper further. First, you need to integrate 'Research method' and 'Sources of data' into a solid paragraph. In this section, you can indicate what kinds of documents you used and how you processed data. Second, the Introduction section is now quite long and many subsections. I suggest that you cut off this section and elaborate on what research questions will be addressed in this article. What are the main research objectives? Third, please reconsider structuring the paragraph in the results sections. In each statement, you need to add more arguments everywhere you refer to statistics and historical evidence. Last but not least, you should employ a professional proofreader to thoroughly check the accuracy of word choices and the clarity of arguments.

 I hope this helps and look forward to reading your revisions soon. Good luck!

Is the work clearly and accurately presented and does it cite the current literature?

Partly

If applicable, is the statistical analysis and its interpretation appropriate?

Partly

Are all the source data underlying the results available to ensure full reproducibility?

Partly

Is the study design appropriate and is the work technically sound?

Partly

Are the conclusions drawn adequately supported by the results?

Partly

Are sufficient details of methods and analysis provided to allow replication by others?

Partly

Reviewer Expertise:

Vietnamese studies, Vietnamese history, contemporary Vietnam, Southeast Asian studies.

I confirm that I have read this submission and believe that I have an appropriate level of expertise to confirm that it is of an acceptable scientific standard, however I have significant reservations, as outlined above.

# Reviewer report: Reviewer response for version 2

**DOI:** 10.5256/f1000research.188699.r421399

**Published:** 2026-01-12

**Authors:** Ha Trieu Huy

**Affiliations:** 1 Vietnam National University, Ho Chi Minh City, Vietnam

The document investigates how the nascent DRV government, led by Ho Chi Minh, utilized diplomacy as a "sharp weapon" to navigate a power vacuum following the collapse of the Japanese Empire. Facing internal exhaustion from famine and external threats from French, Chinese Nationalist, and Allied forces, the DRV employed a "balancing" diplomatic approach to secure legitimacy and buy time for domestic consolidation. The paper concludes that while full-scale war was ultimately unavoidable, the diplomatic efforts of 1945–1946 established the foundational principles of Vietnam’s modern foreign policy: independence, flexibility, and multilateralism.

 However, the author(s) seem unsuccessful to address research questions raised in the first section of this article. The DRV diplomacy soon after the end of the Second World War has been over-explored in historical scholarship. The author, however, does not bring fresh arguments to renew a long-standing perception and provide new scientific evidence. While stating that ‘socio-political and cultural transformations in Vietnamese society that originate in 1945 and were rooted in early 19th-century dynamics’, this article has a superficial ground of these elements across the paper. The evolution of arguments seem unclear and unlogical to lead to research questions. The introduction is now like a collection of historical events that are familiar with general readers.

 Besides, literature review is fairly short and that was a brief section of work statistics rather than a decent paragraph of critical review. Although the author indicated that historical scholarship lacks an integrated analysis that “(i) combines in-depth domestic and international sources; (ii) links discourse analysis with strategic analysis through a chronological examination of diplomatic moves; and (iii) situates Vietnam in systematic comparison with other postcolonial states in order to highlight its specificities.” However, the author failed to address them in this paper. I don’ know why the author indicated  three questions in this section. Are they relevant to research objectives mentioned above? The concept of ‘post-colonial’ should be cleared further.

 In the methodology section, I am confused as the author mentioned ‘historical reconstruction’ method. What is this method? Did it work in this paper as I did’t see any ‘reconstruction’ across the paper. To address it, the author MUST get access to archives that preserved by the "victors" or the literate elite. This creates "archival silences" regarding the experiences of common soldiers, women, or marginalized groups during the 1945–1946 period. Also, the author referred to ‘Fairclough’s Discourse Analysis (CDA)”. While Fairclough’s three-level model (text, discursive practice, social practice) is powerful for revealing power dynamics, did the author know that is often criticized for being "politically committed." A researcher might "find" the power structures or ideologies they expect to see (e.g., assuming the DRV's use of "democracy" was purely strategic rather than aspirational). If the researcher focuses too much on the text (e.g., the 1945 Declaration), they might overlook the social practice—the fact that most of the population was illiterate at the time and experienced "independence" through local action rather than through the nuances of high-level diplomatic language. Besides, Historical Comparison is mentioned, but it doesn’t work effectively in this paper. : Choosing Indonesia and India as "successful" or "comparable" examples might ignore other failed revolutionary movements of the same era that would provide a different perspective on "survival diplomacy."

 More worryingly, the result, which is the heart of this article, seems unsatisfactory. Many paragraphs are merely narrative of what the public knew about the Vietnamese history. Old sources are benefited, but they didn’t help renew a concept of historical scholarship. The author lacks in-depth analysis and assessment how those events worked and to what extent they were effective. The author is suggested to refer to a diversity of secondary sources written by English to make balance for the paper’s stance. The paper risks portraying every decision as a masterstroke of "principled flexibility." In reality, many results of that era—such as the March 6 Preliminary Agreement—were highly controversial at the time and nearly led to a collapse of internal support for the Viet Minh. By focusing on the "foundation for modern policy," the results may gloss over the fact that these diplomatic efforts ultimately failed to prevent a decade-long bloody war. Besides, the paper heavily utilizes Fairclough’s discourse analysis to show how the DRV used terms like "independence" and "equality" to gain legitimacy. There is a "gap" in the results between language and geopolitical reality. While the paper proves the DRV used internationalist language effectively, the material result was that no major power (not even the Soviet Union at that time) formally recognized the DRV in 1945–1946. The results emphasize "discursive legitimacy" but fail to sufficiently address why this discourse failed to translate into actual diplomatic recognition until 1950.

 Besides, the comparison appears superficial in the results. For example, India’s "survival" was facilitated by a structured British withdrawal, whereas Vietnam’s was a violent struggle against a returning colonial power. By grouping these together under a single umbrella of "survival diplomacy," the paper loses the specific nuances of why Vietnam’s outcome (a 30-year war) was so radically different from India’s (partition and Commonwealth membership). The paper treats "the DRV" as a monolithic actor. It lacks data on how local committees or Southern resistance leaders viewed these diplomatic concessions. The result—that diplomacy helped "consolidate domestic institutions"—might be inaccurate if those very diplomatic concessions (like allowing French troops back into the North) caused fragmentation or anger among the revolutionary grassroots.

 In conclusion, while the paper addresses a pivotal moment in Vietnamese history—the diplomatic struggle for survival between 1945 and 1946—it ultimately falls short of making a significant original contribution to the field. The work functions more as a historical narrative of well-known events than a rigorous academic analysis. Its primary failings lie in the disconnect between its ambitious theoretical framework and its actual execution. To reach a publishable standard, the author must move beyond a descriptive collection of familiar events. This requires:

1. Deepening the Archival Research: Accessing primary sources that represent non-elite or dissenting perspectives to address "archival silences."
2. Critical Distance: Evaluating diplomatic decisions (like the March 6 Preliminary Agreement) not just as successes, but as potential sources of internal fragmentation.
3. Synthesizing Method with Evidence: Ensuring that the discourse analysis and comparisons actually drive the argument forward rather than serving as mere academic window dressing.

Without these revisions, the paper remains a summary of established historical knowledge rather than a critical advancement in the study of post-colonial diplomacy.

Is the work clearly and accurately presented and does it cite the current literature?

Partly

If applicable, is the statistical analysis and its interpretation appropriate?

Partly

Are all the source data underlying the results available to ensure full reproducibility?

Partly

Is the study design appropriate and is the work technically sound?

Partly

Are the conclusions drawn adequately supported by the results?

Partly

Are sufficient details of methods and analysis provided to allow replication by others?

Partly

Reviewer Expertise:

Vietnamese studies, Vietnamese history, contemporary Vietnam, Southeast Asian studies.

I confirm that I have read this submission and believe that I have an appropriate level of expertise to confirm that it is of an acceptable scientific standard, however I have significant reservations, as outlined above.

# Reviewer report: Reviewer response for version 2

**DOI:** 10.5256/f1000research.188699.r433997

**Published:** 2025-12-27

**Authors:** Pedro Emanuel Mendes

**Affiliations:** 1 Universidade Nova de Lisboa, Lisboa, Portugal

I agree with the arguments made by my fellow reviewers regarding the difficulties and organizational gaps in the article, as well as its overly apologetic tone.

 There is also the general problem of several quotations in quotation marks without proper references.

 Therefore, I will attempt to offer new recommendations.

 This article analyzes the foreign policy of modern independent Vietnam after the communist revolution and under the leadership of Ho Chi (1945-1946).

 The topic is of interest, but there are several methodological gaps that must be addressed.

 The Introduction needs to identify the problem being analysed and the questions to which it seeks answers.

 For example, this article is about x, and the problem it aims to analyse is Y. To this end, we introduce questions 1, 2, and 3 (possibly those posed by the author).

 Contrary to what has been incorrectly stated, the theme and issue of the article are not: **“This paper examines the socio-political and cultural transformations in Vietnamese society that originate in 1945 and were rooted in early 19th-century dynamics”.**

 Even the problem itself seems to be there…\But, as always, the author has difficulty clearly and objectively identifying it in a single sentence.

 Another issue is the author's failure to engage with theoretical works from Foreign Policy Analysis (FPA) that could help improve the article.

 More than the historical facts—which are already known—it is important for the author to better problematize these facts, particularly in characterizing post-colonial foreign policies that, although communist and revolutionary (from an internal point of view); externally they attempt to play by the rules of liberal international norms to gain international legitimacy and political independence.

 This is essentially the best idea of this study, but it should be clearer and more thoroughly developed.

 Additionally, a section on regional and international historical contexts linked to post-colonial and international communist movements could help place Ho Chi Minh’s Vietnam in context and distinguish it from other countries.

 Another good idea is to characterise Ho Chi Minh’s ideas and worldview and try to connect them to the ideas and practices of Vietnam’s foreign policy. However, this idea requires further development.

 In the conclusion, the author should objectively answer the questions posed regarding the problems.

Is the work clearly and accurately presented and does it cite the current literature?

No

If applicable, is the statistical analysis and its interpretation appropriate?

Not applicable

Are all the source data underlying the results available to ensure full reproducibility?

Partly

Is the study design appropriate and is the work technically sound?

Partly

Are the conclusions drawn adequately supported by the results?

No

Are sufficient details of methods and analysis provided to allow replication by others?

No

Reviewer Expertise:

International Relations; Foreign Policy Analysis; Southeastasia;

I confirm that I have read this submission and believe that I have an appropriate level of expertise to state that I do not consider it to be of an acceptable scientific standard, for reasons outlined above.

# Reviewer report: Reviewer response for version 2

**DOI:** 10.5256/f1000research.188699.r420839

**Published:** 2025-10-08

**Authors:** Bama andika putra

**Affiliations:** 1 Schoo, University of Bristol School of Sociology Politics and International Studies, Bristol, England, UK

Thank you for the revisions. I no longer have any concerns and propose the article to be indexed.

Is the work clearly and accurately presented and does it cite the current literature?

Yes

If applicable, is the statistical analysis and its interpretation appropriate?

Not applicable

Are all the source data underlying the results available to ensure full reproducibility?

Yes

Is the study design appropriate and is the work technically sound?

Yes

Are the conclusions drawn adequately supported by the results?

Partly

Are sufficient details of methods and analysis provided to allow replication by others?

Partly

Reviewer Expertise:

International relations

I confirm that I have read this submission and believe that I have an appropriate level of expertise to confirm that it is of an acceptable scientific standard.

# Reviewer report: Reviewer response for version 1

**DOI:** 10.5256/f1000research.183642.r400250

**Published:** 2025-09-09

**Authors:** Kiet Le Hoang

**Affiliations:** 1 Yersin University, Dalat, Vietnam

I would like to thank the Editorial Board for their trust in my expertise. This manuscript holds value and significance for Vietnamese history and helps the world better understand the foreign policy of the Democratic Republic of Vietnam during the period immediately after seizing power from the Japanese Empire in the August Revolution of 1945. After reading this manuscript, I found that its main objective is to explain the difficult situation of young Vietnam under tremendous pressure from external powers (1945-1946), and having to adjust foreign policy very flexibly toward French colonialists and Chiang Kai-shek's army to protect the nascent independence. 

 First, I comment on the Introduction section of the article. The article mentions the historical context, but does not explain why this topic is important and needs to be studied? To my knowledge, this topic has been researched by many famous historians in Vietnam, and I do not see what new contribution this article offers compared to these existing publications. Moreover, the main arguments and research questions posed are quite vague, the argumentative presentation in the Introduction is inadequate with unreasonable line breaks, inappropriate use of subheadings, and lacks scientific rigor. 

 Second, this article belongs to the field of History, where typically the Literature Review section is very important for History to establish the author's academic foundation on the research topic, identify existing gaps in current literature, and assess the reliability of historical sources, especially given the intense academic debates characteristic of the History discipline. However, the article lacks this section, making it very weak in terms of scientific structure and research purpose. 

 Third, regarding methodology. The methodology section is scientifically weak, with many methods mentioned but their implementation and application in the Results section are absent and not discussed anywhere, failing to demonstrate the "dialectical" relationship between Vietnam's revolutionary objectives and global power structures. I do not see any comparison between decolonization movements of Vietnam and India, Indonesia as mentioned. The Marxist-Leninist principles are not presented and proven with evidence in the Results section.

  Fourth, regarding the results and discussion section. The content presented is nothing new and has been analyzed extensively, the terminology does not meet international standards, using unscientific language and reading more like propaganda articles than scientific research. There is insufficient empirical data to defend the arguments, the lessons learned section is unnecessary, and the expression of content is inadequate for publication. Most importantly, it lacks academic debate, lacks objectivity in historical research, mainly focusing on praise, oversimplifying success to "wise leadership," and contains no new findings. 

 Fifth, regarding the conclusion. It only summarizes what was already said in the Results section, with no new synthetic analysis or deep insights, repeating the old formula: "under the leadership of the Party and President Ho Chi Minh." The conclusion does not answer the research questions initially posed, contains no critical evaluation, lacks specific comparison with similar cases worldwide. It fails to indicate new research findings, does not propose future research directions, and lacks positioning within the international research context. Finally, regarding the overall structure, the article lacks balance between sections and lacks materials from relevant countries like France and China to enhance the academic debate of the article.

Is the work clearly and accurately presented and does it cite the current literature?

Partly

If applicable, is the statistical analysis and its interpretation appropriate?

No

Are all the source data underlying the results available to ensure full reproducibility?

Partly

Is the study design appropriate and is the work technically sound?

No

Are the conclusions drawn adequately supported by the results?

No

Are sufficient details of methods and analysis provided to allow replication by others?

No

Reviewer Expertise:

International Relations

I confirm that I have read this submission and believe that I have an appropriate level of expertise to state that I do not consider it to be of an acceptable scientific standard, for reasons outlined above.

# Response

**Published:** 2025-09-23

**Authors:** Quoc Dung Mai

**Affiliations:** Ho Chi Minh City University of Industry and Trade, Vietnam

First of all, I would like to thank you for reviewing my article. I sincerely acknowledge your comments, and within my capacity and the accessible sources, I have tried to address some of your suggestions, such as: the article now discusses the complexity of the international situation after World War II (at a descriptive level rather than in-depth analysis) with specific comparisons to other postcolonial cases such as Indonesia and India; the originality is more clearly demonstrated through a comparative approach combining discourse analysis with strategic analysis; the literature review and methodological application are presented more explicitly, with additional empirical evidence incorporated to strengthen the arguments; and certain points are presented with greater clarity, with more firmly grounded reasoning, standardized terminology, correction of propagandistic tone, and a conclusion that provides synthesized analysis and positions the study within academic debates.

 (Please read the revised version, which will be published in the near future.)

 I hope you will accept the article in its revised version. With sincere thanks.

# Reviewer report: Reviewer response for version 1

**DOI:** 10.5256/f1000research.183642.r398620

**Published:** 2025-09-03

**Authors:** Tran Xuan Hiep

**Affiliations:** 1 The University of Da Nang, Đà Nẵng City, Vietnam

Title: Vietnam's Foreign Policy (1945-1946): Proactive Amidst Fragile Independence

**Pro:**

 - The article provides a valuable commentary on Vietnam's diplomatic activities from 1945 to 1946, highlighting relevant lessons for modern Vietnamese diplomacy.

 - The author demonstrates extensive use of sources, citing various declassified archives, Party resolutions, works by Ho Chi Minh, and both Vietnamese and international academic research.

 - The chronological structure is clear and logical, organizing the content into distinct sections: context, methods, results, discussion, and lessons learned.

**Cons:**

 - The article lacks analysis of the global context, particularly the emerging Cold War, and fails to discuss U.S. policy on communism and French foreign policy in the post-WWII period.

 - There is a lack of evidence to support the distinction between “friends and enemies” as discussed in the section, “The Lesson of Distinguishing Enemies,” especially when relating it to Party documents from the Doi Moi (Renovation) period.

 - The author does not analyze how the Party and State of Vietnam have applied the lesson of "making concessions at the right time, with limits, and with principles" to contemporary foreign policy, leaving a significant gap in the analysis of this lesson.

 - Although the author states a Marxist-Leninist research method, the reviewer questions its application, as the references provided do not appear to fully reflect this approach. Furthermore, while the author claims to have used declassified archival materials (e.g., Party documents, governmental texts, diplomatic records), these specific sources are not listed in the references, making it difficult to verify the claims.

Is the work clearly and accurately presented and does it cite the current literature?

No

If applicable, is the statistical analysis and its interpretation appropriate?

Not applicable

Are all the source data underlying the results available to ensure full reproducibility?

Partly

Is the study design appropriate and is the work technically sound?

No

Are the conclusions drawn adequately supported by the results?

Partly

Are sufficient details of methods and analysis provided to allow replication by others?

No

Reviewer Expertise:

History, Political, Culture, International Relations

I confirm that I have read this submission and believe that I have an appropriate level of expertise to state that I do not consider it to be of an acceptable scientific standard, for reasons outlined above.

# Response

**Published:** 2025-09-23

**Authors:** Quoc Dung Mai

**Affiliations:** Ho Chi Minh City University of Industry and Trade, Vietnam

First of all, I would like to thank you for reviewing my article. I sincerely acknowledge your comments, and within my capacity and the available sources, I have tried to address some of your suggestions, such as: clarifying the theoretical framework and the application of methods; including discussion of the complexity of the international situation after World War II (at a descriptive level rather than in-depth analysis); and demonstrating more clearly the originality of the study through a comparative approach that combines discourse analysis with strategic analysis.

 (Please read the revised version, which will be published in the near future.)

 I hope you will accept the article in its revised version. With sincere thanks.

# Reviewer report: Reviewer response for version 1

**DOI:** 10.5256/f1000research.183642.r405277

**Published:** 2025-09-02

**Authors:** Bama andika putra

**Affiliations:** 1 Schoo, University of Bristol School of Sociology Politics and International Studies, Bristol, England, UK

The article provides a historic overlook on Vietnam's foreign policy between 1945 and 1946. Several comments for revisions:

 1. Analytical framework: The inclusion of historical method, logical method, intertextual analysis, critical discourse analysis, can also be perceived as this article's main source of difficulty. First, it was not made clear what specific method/ points of analysis are being used within the text. Second, there needs to be clarification as to how each of the method/ analysis are utilized within the texts. Do they complement one another? Or do they act to provide different points of perspectives?

 2. Empirical investigation: The article does cover most of the points outlined as the aim of the study. However, I believe there are other points to emphasize in the early years of Vietnam's struggle. I would consider incorporating data that discusses regional contexts, such as, how the region influenced (or not) the foreign policies undertaken during this time period.

 3. Novelty: This article needs to provide the explanation as to what precisely is it contributing towards the relevant discourses. Is it original in a sense that no authors in the past have evaluated the issues of Vietnam's independence? Or is it original due to the incorporation of different methods to analyze the texts? On that point, I believe that this can also be highlighted in the conclusion section.

Is the work clearly and accurately presented and does it cite the current literature?

Yes

If applicable, is the statistical analysis and its interpretation appropriate?

Not applicable

Are all the source data underlying the results available to ensure full reproducibility?

Yes

Is the study design appropriate and is the work technically sound?

Yes

Are the conclusions drawn adequately supported by the results?

Partly

Are sufficient details of methods and analysis provided to allow replication by others?

Partly

Reviewer Expertise:

International relations

I confirm that I have read this submission and believe that I have an appropriate level of expertise to confirm that it is of an acceptable scientific standard, however I have significant reservations, as outlined above.

# Response

**Published:** 2025-09-23

**Authors:** Quoc Dung Mai

**Affiliations:** Ho Chi Minh City University of Industry and Trade, Vietnam

First of all, I would like to thank you for reviewing my article. I sincerely acknowledge your comments, and within my capacity and the available sources, I have tried to address some of your suggestions, such as: clarifying the analytical framework and the application of methods (historical, logical, intertextual, discourse analysis) and their complementary relationship; providing a clearer explanation of the article’s contribution to the relevant discourse; and revising the conclusion to align more closely with the study’s findings.

 (Please read the revised version, which will be published in the near future)

 With sincere thanks.
